# Maize Terpene Synthase 8 (ZmTPS8) Contributes to a Complex Blend of Fungal-Elicited Antibiotics

**DOI:** 10.3390/plants12051111

**Published:** 2023-03-01

**Authors:** Evan V. Saldivar, Yezhang Ding, Elly Poretsky, Skylar Bird, Anna K. Block, Alisa Huffaker, Eric A. Schmelz

**Affiliations:** 1 Department of Cell and Developmental Biology, University of California at San Diego, San Diego, CA 92093, USA; 2 Department of Plant Biology, Carnegie Institution for Science, Stanford University, Palo Alto, CA 94305, USA; 3 Environmental Genomics and Systems Biology, Lawrence Berkeley National Laboratory, Berkeley, CA 94720, USA; 4 Chemistry Research Unit, U.S. Department of Agriculture-Agricultural Research Service, Center for Medical, Agricultural and Veterinary Entomology, Gainesville, FL 32608, USA

**Keywords:** maize specialized metabolism, defense, sesquiterpenoids, *Fusarium*, terpene synthase

## Abstract

In maize (*Zea mays*), fungal-elicited immune responses include the accumulation of terpene synthase (TPS) and cytochrome P450 monooxygenases (CYP) enzymes resulting in complex antibiotic arrays of sesquiterpenoids and diterpenoids, including α/β-selinene derivatives, zealexins, kauralexins and dolabralexins. To uncover additional antibiotic families, we conducted metabolic profiling of elicited stem tissues in mapping populations, which included B73 × M162W recombinant inbred lines and the Goodman diversity panel. Five candidate sesquiterpenoids associated with a chromosome 1 locus spanning the location of *ZmTPS27* and *ZmTPS8*. Heterologous enzyme co-expression studies of ZmTPS27 in *Nicotiana benthamiana* resulted in geraniol production while ZmTPS8 yielded α-copaene, δ-cadinene and sesquiterpene alcohols consistent with *epi*-cubebol, cubebol, copan-3-ol and copaborneol matching the association mapping efforts. ZmTPS8 is an established multiproduct α-copaene synthase; however, ZmTPS8-derived sesquiterpene alcohols are rarely encountered in maize tissues. A genome wide association study further linked an unknown sesquiterpene acid to ZmTPS8 and combined ZmTPS8-ZmCYP71Z19 heterologous enzyme co-expression studies yielded the same product. To consider defensive roles for ZmTPS8, in vitro bioassays with cubebol demonstrated significant antifungal activity against both *Fusarium graminearum* and *Aspergillus parasiticus*. As a genetically variable biochemical trait, ZmTPS8 contributes to the cocktail of terpenoid antibiotics present following complex interactions between wounding and fungal elicitation.

## 1. Introduction

As critical resources for food, feed and fuel, grain crops merit close investigation of their precise biochemicals that function as mediators of complex interactions underlying plant resilience [1]. Despite considerable efforts, many plant specialized metabolites commonly go undetected due to highly conditional biosynthesis driven by precise combinations of the biotic environment, the abiotic environment and plant genetic variation [2]. Given the challenge in understanding existing genetic resources underlying crop plant resilience, a continued research goal is to connect genotypes to measurable phenotypes [3,4]. In nearly all plant models, diverse arrays of terpenoids underlay a significant component of biochemical complexity [5]. Encoded by medium-sized plant gene families, terpene synthases (TPS) drive the complex production of volatile hydrocarbon olefins and often mono-oxygenated products with extraordinary structural variability that can function directly or serve as precursors in protective pathways with further expansive biochemical complexity [5,6]. In maize (*Zea mays*), commonly encountered volatile terpenes include monoterpenes (C_10_), sesquiterpenes (C_15_), modified homoterpenes (C_11_, C_16_) and diterpenes (C_20_) [7]. The conversion of precursors such as geranyl pyrophosphate (GPP), farnesyl pyrophosphate (FPP) and geranyl geranyl pyrophosphate (GGPP) into mono-, sesqui- and diterpene products, respectively, is catalyzed by TPS which commonly results in diverse hydrocarbon olefins and mono-oxygenated products [8]. An exception in maize is ZmTPS17, termed eudesmanediol synthase (ZmEDS), which directly produces a dioxygenated sesquiterpenoid, namely the eudesmane-2,11-diol from FPP [9].

Volatile plant TPS products serve diverse biological roles including the attraction of pollinators to flowers, attraction of natural enemies to arthropod pests and underlay antimicrobial defenses [10,11,12]. In maize, leaf and root feeding herbivores, such as lepidoptera caterpillars and rootworm (*Diabrotica* spp.) beetle larvae, elicit the production of terpenes that serve as indirect defense mechanisms and ecological signals in the attraction of natural enemies to the attacking pests [10,13,14]. Of 43 TPS present in the maize W22 genome [15], approximately 30 ZmTPS have been characterized in efforts to better understand biochemical, physiological and ecological functions [9,16,17,18]. Key maize genes with established indirect defense roles are the α-bergamotene/β-farnesene synthase (ZmTPS10) and the *E*-β-caryophyllene synthase (ZmTSPS23) [14,19,20]. Of approximately 30 commonly detectable maize terpene volatiles, 12 TPS are largely responsible for their production [7]. *ZmTPS* transcript accumulation and elicited volatile production are often examined in the biological context of responses to mechanical wounding, insect herbivory, herbivore associated molecular patterns (HAMPs) and damage-associated molecular patterns (DAMPs) [19,20,21,22,23]. In maize, the detection of strong positive *TPS* transcriptional responses to insect herbivory are commonly used in part to assign probable roles in plant indirect defense responses more generally [24].

Beyond roles as volatile signals, an increasing number of non-volatile TPS products have been recently discovered following microbial challenge. So far, the maize α/β-selinene synthase (ZmTPS21), four β-bisabolene/β-macrocarpene synthases (Zx1 to Zx4), an *ent*-copalyl diphosphate synthase (anther ear 2: ZmAn2), *ent*-isokaurene synthase (kaurene synthase-like 2: ZmKSL2) and a dolabradiene synthase (ZmKSL4) have been demonstrated to display transcript accumulation following fungal elicitation and drive the production of non-volatile antibiotic families termed α/β-costic acids [3], zealexins [17], kauralexins [18,25] and dolabralexins [26,27], respectively. To date, each pathway family has been demonstrated to generate predominant end-products which at physiologically relevant levels display significant activities against pathogenic fungi in the genus *Fusarium* and others. Common oxidative enzymes acting in early maize TPS antibiotic production are cytochrome P450 monoxygenases (CYP) in the ZmCYP71Z family, namely, ZmCYP71Z16, ZmCYP71Z18 and ZmCYP71Z19 which are colocalized in a chromosome 5 gene cluster [17]. Currently, ZmCYP71Z19 appears to not be involved in kauralexin biosynthesis and displays partial specificity to sesquiterpene precursors [17]. Both ZmCYP71Z18 and ZmCYP71Z16 are active in diterpenoid biosynthesis [18,26]; however, all 3 ZmCYP71Z members oxidize β-macrocarpene and β-bisabolene to produce A and D-series zealexins [17]. An important aspect of maize terpenoid antibiotic biosynthesis is the presence of duplicated and partially functionally divergent ZmCYP71Z family enzymes which retain significant substrate promiscuity to create an hourglass shaped biosynthetic pathway capable of using diverse substrates to generate complex defensive blends [17]. Thus, existing pathway knowledge strongly suggests the potential for additional modular and genetically variable TPS enzymes that are likely to further contribute to maize antibiotic production [17].

To date, analyses of fungal-elicited maize tissues have revealed the presence of at least 35 terpenoids not readily detected in healthy tissues [3,17,18,26]. In this study, we sought to consider additional sesquiterpenoids present in fungal-elicited tissues by examining diverse maize inbred lines. A list of B73 RefGen_v2_v3_v4 gene IDs and protein IDs with relevance to maize terpenoid biosynthesis is provided (Table S1). Using forward genetics enabled by metabolite-based association mapping approaches, we identified *ZmTPS* gene candidates linked to complex fungal-elicited maize terpenoids. Specifically, we combined targeted gas chromatography mass spectrometry (GC/MS) metabolic profiling with an association analysis of a B73 × M162W recombinant inbred lines (RILs) and the Goodman diversity panel for a genome-wide association study (GWAS) to associate five previously undetected sesquiterpenoids with a locus on chromosome 1. Two *ZmTPS* genes, namely *ZmTPS27* (uncharacterized) and *ZmTPS8*, encoding an α-copaene/germacrene D synthase spanned the locus and were assayed using *Agrobacterium*-mediated heterologous enzyme co-expression in *Nicotiana benthamiana*. Expression of ZmTPS27 resulted in geraniol production while ZmTPS8 yielded α-copaene, δ-cadinene and four additional sesquiterpene alcohols consistent with *epi*-cubebol, cubebol, copan-3-ol and copaborneol. ZmTPS8 exists as an established multiproduct α-copaene synthase with a role in indirect defense against herbivory [28]; however, production of more diverse ZmTPS8-derived sesquiterpenoids and their role in additional plant-biotic interactions have not been closely examined. Association analyses further connected an unknown sesquiterpene acid with ZmTPS8 and product synthesis was confirmed via heterologous co-expression of ZmTPS8 and ZmCYP71Z19 in *N. benthamiana*. In vitro antifungal assays with the ZmTPS8 product cubebol and the promiscuous activity of ZmCYP71Z19 on TPS8 products are consistent with complex biosynthetic pathway interactions and an additive role for ZmTPS8 in stem defenses against fungal pathogens.

## 2. Results

### 2.1. Identification of Previously Undetected Maize Sesquiterpenoids in Stems following Elicitation

**Figure 1:**
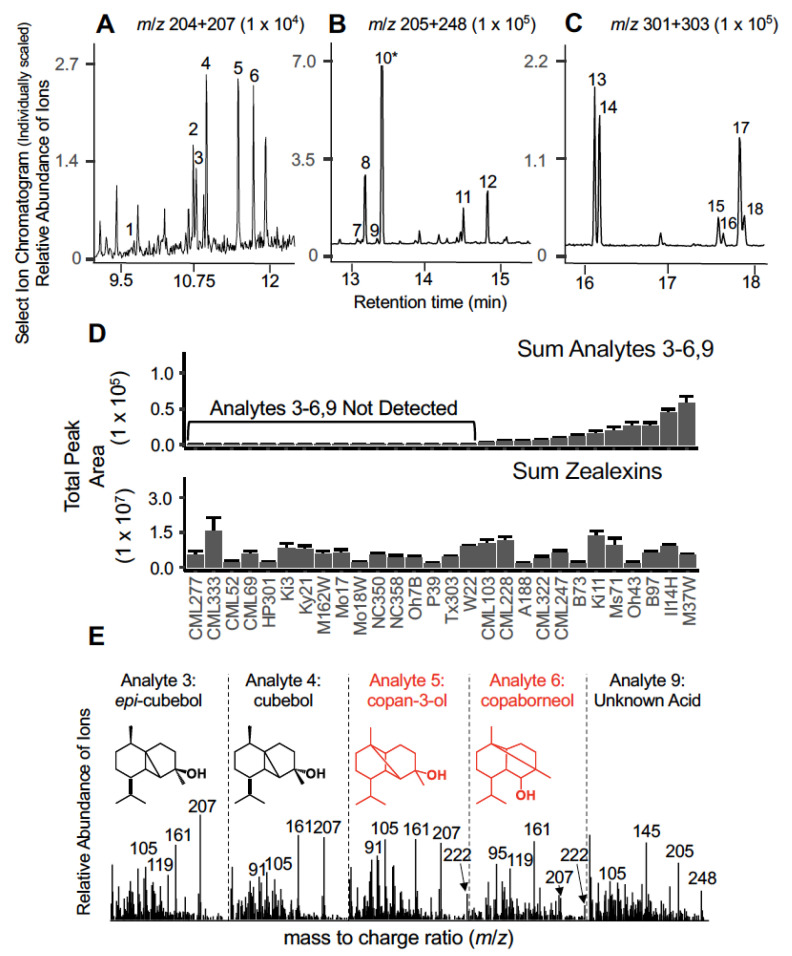
*Maize stems accumulate four sesquiterpene alcohols following fungal elicitation in a genotype-dependent manner*. (**A**–**C**) GC/MS Select Ion Chromatograms (SIC) of M37W stem tissue 3 days after wounding and fungal elicitation. Previously established representative maize analytes include (1) α-copaene, (2) β-macrocarpene, (7) Zealexin D2, (8) Zealexin D1, (10) Zealexin A1, (11) Zealexin A2, (12) Zealexin A3, (13) Kauralexin A1, (14) Kauralexin B1, (15) Kauralexin A2, (16) Kauralexin B2, (17) Kauralexin A3 and (18) Kauralexin B3. Previously undetected oxygenated sesquiterpenoids (analytes 3, 4, 5, 6 and 9) are the focus of this study. Individual plots represent distinct SIC traces, consistent with different molecular structures (**A**) *m*/*z* 204 + 207 (sesquiterpene olefins and sesquiterpene alcohol fragment ions), (**B**) *m*/*z* 205 + 248 (sesquiterpene acid methyl ester derivatives and fragments) and (**C**) *m*/*z* 301 + 303 (diterpene acid methyl ester derivative fragment ions). (**D**) Average (*n* = 3, +SEM) peak areas across fungal-elicited stem tissues for Mo17, W22, A188, B73 and inbreds used from the nested association mapping (NAM) population for the unknown sesquiterpenoids (sum of analytes 3, 4, 5, 6 and 9) and previously established zealexins (sum of analytes 2, 7, 10, 11 and 12). (**E**) GC/MS spectral library matches and corresponding chemical structures for analyte 3, 4, 5, 6 and 9, assigned by cross referencing with *Piper cubeba* essential oil standards and literature searches. Analytes in black (#3, *epi*-cubebol and #4, cubebol) are supported by retention time matches and *Piper cubeba* reference spectra. Analytes drawn in red (#5, copan-3-ol and #6, copaborneol) are supported based on similarity to published reference spectra [30]. Analyte #9 is consistent with unknown sesquiterpene acid (analyzed as a methyl ester derivative). M37W reference EI spectra for each analyte are provided and correspond to the GC/MS SIC presented in panel A.

In maize, fungal challenge activates synthesis of complex terpenoid blends [3,17,18,26]. To further understand maize biochemical diversity and the underlying genetic basis, we performed targeted metabolic profiling on stems of B73, W22, Mo17 and the nested association mapping (NAM) inbred parental lines [29]. Stems were treated with a heat-killed *Fusarium venenatum* to screen for metabolomic diversity following fungal elicitation. Of the 28 inbred lines examined, 12 accumulated 5 previously undetected sesquiterpenoid candidates (analytes 3, 4, 5, 6 and 9) (Figure 1A) co-present with representative sesquiterpenoid zealexins (Figure 1B) [17] and kauralexins (Figure 1C) [18]. A low abundance analyte was α-copaene (analyte 1) which chromatographs much earlier than the established zealexin precursor β-macrocarpene (analyte 2) (Figure 1A). Despite co-presence, the overall accumulation patterns of the unknown sesquiterpenoids did not track zealexin accumulation (Figure 1D). The specified unknown candidate sesquiterpenoids (analytes 3, 4, 5 and 6) displayed either suspected parent *m*/*z* 222 ions or common diagnostic *m*/*z* 207 fragment ions consistent with sesquiterpene alcohols (Figure 1E). Electron-ionization (EI) mass spectral searches of libraries and published spectra from established TPS catalyzing the production of germacrene D intermediates [30] enabled identification of the following sesquiterpene alcohols, *epi*-cubebol (analyte 3), cubebol (analyte 4), copan-3-ol (analyte 5) and copaborneol (analyte 6) (Figure 1E). Analytes 3 and 4 were further supported as *epi*-cubebol and cubebol, respectively, by comparison of retention times and EI mass spectra of an authentic sample of *Piper cubeba* essential oil (Figure S1). In contrast to confirmation of *epi*-cubebol and cubebol with authentic standards, the tentative identities of copan-3-ol and copaborneol are based on comparatively unique EI spectra matches from a multiproduct TPS that also produces cubebol [30]. Analyte 9, with a parent ion of 248 *m*/*z*, represents a further unknown consistent with a previously undetected sesquiterpene acid displaying a representative chromatographic retention time after ZD1, yet before ZA1 following methyl ester derivatization [17].

### 2.2. Association Mapping Using the B73 × M162W Recombinant Inbred Line (RIL) Population Identifies a Locus on Chromosome 1 Controlling Oxygenated Maize Sesquiterpenoids

**Figure 2:**
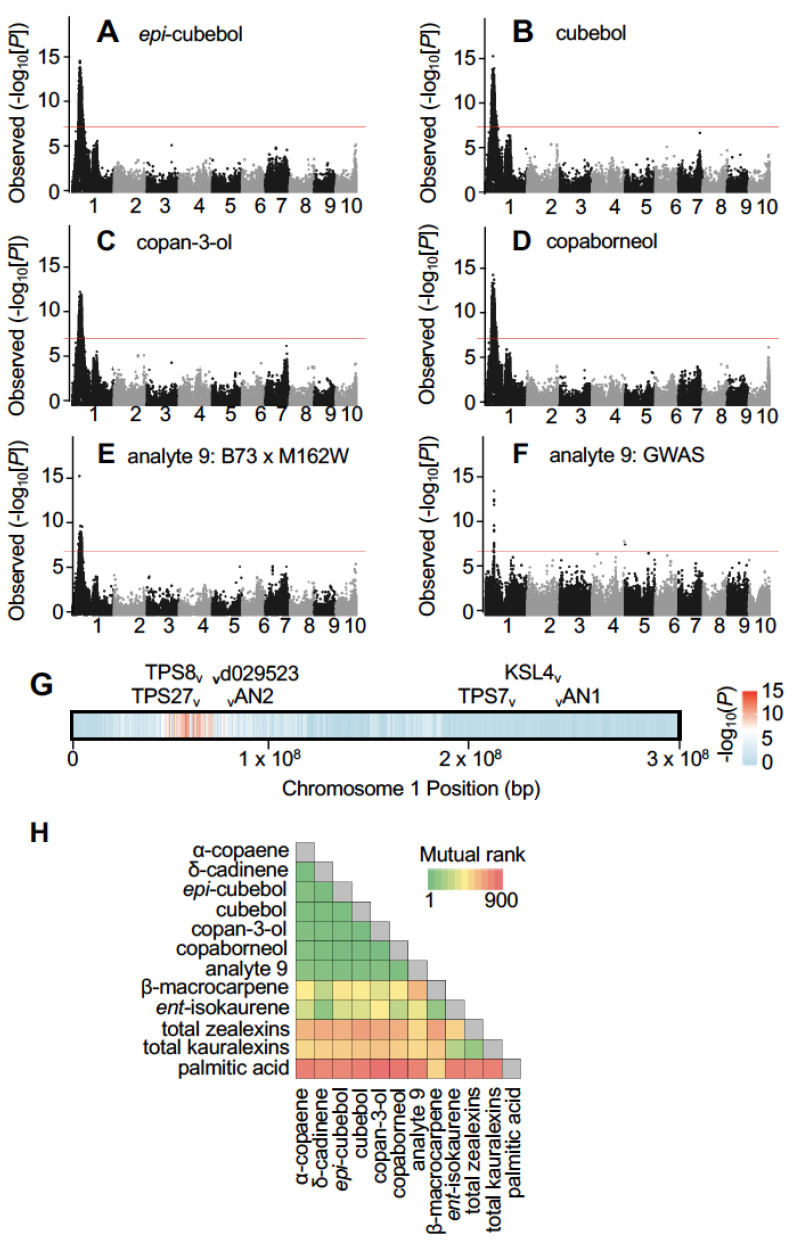
The co-occurrence of 5 rarely detected maize sesquiterpenoids is genetically associated with two candidate terpene synthase genes (*ZmTPS*) on Chromosome 1. (**A**–**F**) Metabolite-led association mapping using the abundance of novel oxygenated sesquiterpenes as mapping traits for B73 × M162W RILs (**A**–**E**) and (**F**) a genome-wide association study (GWAS). Manhattan plots are presented for the association analysis (general linear model) using the M162W × B73 NAM RILs following 5 days of fungal elicitation in stems and using (**A**) *epi*-cubebol, (**B**) cubebol, (**C**) copan-3-ol, (**D**) copaborneol and analyte 9 (unknown sesquiterpene acid) levels as traits. Negative log_10_-transformed *p* values are presented on the *y*-axis. The dashed line denotes the 5% Bonferroni-corrected threshold for 139,001 single nucleotide polymorphism (SNP) markers (B73 RefGen_V2). The most statistically significant chromosome 1 SNPs detected for each analyte were as follows: *epi*-cubebol (56558762), cubebol (55505430), copan-3-ol (55505430), copaborneol (55505430) and analyte 9 (unknown sesquiterpene acid, 50708423). (**E**) Manhattan plot of the association analysis (compressed mixed linear model) of analyte 9 in the Goodman diversity panel following 3 days of fungal elicitation. The dashed line denotes the 5% Bonferroni-corrected threshold for 246,477 SNP markers, with the most statistically significant SNP located at position 61,013,646 (B73 RefGen_V2) on chromosome 1. (**G**) Location of the 7 annotated *ZmTPS* genes (B73 RefGen_V2, V4; *ZmTPS27*, AC205502.4_FG004, Zm00001d029139; *ZmTPS8,* GRMZM2G038153, Zm00001d029195; *d029523*, N/A, Zm00001d029523, *ZmAN2*, GRMZM2G044481, Zm00001d029648; *ZmTPS7*, AC217050.4_FG007, Zm00001d032230; *ZmKSL4*, GRMZM2G016922, Zm00001d032858; *ZmAN1*, GRMZM2G081554, Zm00001d032961) residing on B73 chromosome 1, with negative log_10_-transformed *p* values from (A; *epi*-cubebol) represented as a heatmap. (**H**) Metabolite-based Mutual Rank analyses of selected stem analytes in the M162W × B73 NAM RILs including α-copaene, δ-cadinene, *epi*-cubebol, cubebol, copan-3-ol, copaborneol, analyte 9, β-macrocarpene, *ent*-isokaurene, total zealexins, total kauralexins and palmitic acid.

In maize, forward genetic mapping aided in the identification of biosynthetic genes underlying the production of α/β-costic acids, zealexins and kauralexins [3,17,18]. Upon fungal elicitation, B73 produces modest yet detectable quantities of 5 newly observed oxygenated sesquiterpenes while M162W is completely deficient (Figure 1D). Metabolite presence/absence variation encouraged our use of the B73 × M162W RIL mapping population (Table S2). *Epi*-cubebol, cubebol, copan-3-ol and copaborneol were independently mapped using the B73xM162W RIL population to an identical region on chromosome 1 (Figure 2A–D). The observed sesquiterpene acid, termed analyte 9 (Figure 1B,E) was also mapped to the same locus on chromosome 1 using the B73 × M162W RILs and additionally the Goodman association panel [29] following analysis by a genome-wide association study (GWAS) (Figure 2E,F; Table S2). Our collective association mapping results supported a single significant locus on chromosome 1 spanning two *TPS* genes, namely *ZmTPS27* (AC205502.4_FG004) and *ZmTPS8* (GRMZM2G038153) (Figure 2G; Table S3). To examine and confirm metabolite co-abundance, we performed a Mutual Rank-based analysis [31] on the newly detected sesquiterpenoids from our current study, β-macrocarpene, *ent*-isokaurene, and summed estimates of zealexins, kauralexins and palmitic acid as an unrelated control. With low Mutual Rank values, the terpene olefins α-copaene and δ-cadinene, were highly correlated with the presence of *epi*-cubebol, cubebol, copan-3-ol, copaborneol and analyte 9, an unknown sesquiterpene acid (Figure 2H). Weaker correlations existed between the chromosome 1 associated sesquiterpenoids and established zealexin/kauralexin precursors and end-products, while no meaningful correlations existed with palmitic acid (Figure 2H). Our results support genetic variation and tight co-regulation of an additional subset of maize sesquiterpenoids co-present with established antibiotic pathways.

### 2.3. Heterologous Expression of ZmTPS27 in N. benthamiana Supports Function as a Geraniol Synthase

**Figure 3:**
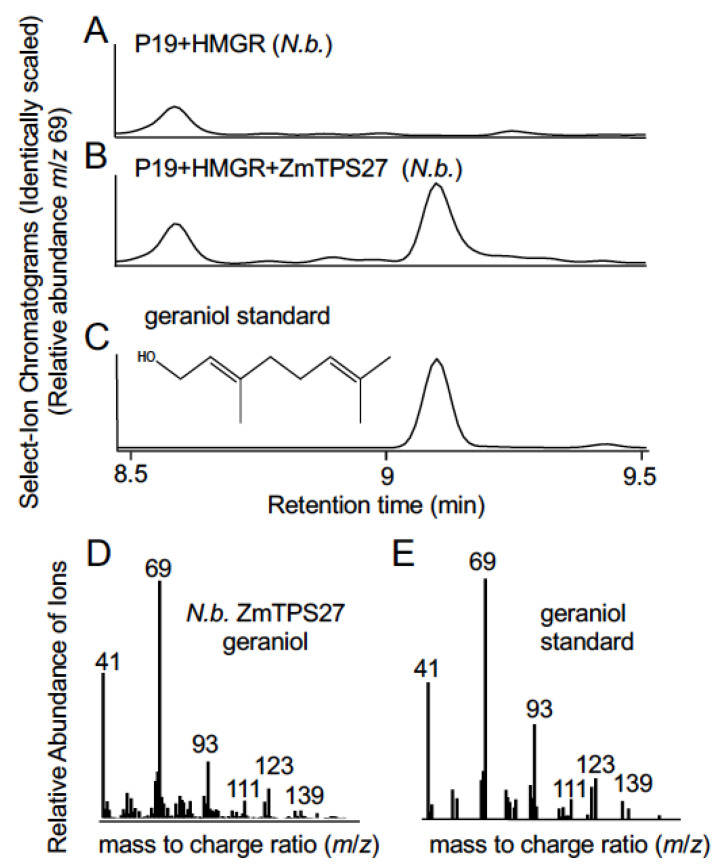
*Heterologous expression of ZmTPS27 in N. benthamiana promotes production of the monoterpene geraniol.* (**A**–**C**) Production of geraniol through heterologous expression of ZmTPS27 in *N. benthamiana*. Identically scaled GC/MS Select Ion Chromatograms (*m*/*z* = 69) are presented, following vapor phase extraction of *N. benthamiana* leaf tissues transiently expressing (**A**) HMGR *+* P19 as a negative control and (**B**) HMGR + P19 + ZmTPS27 to test for terpenoid product accumulation. (**C**) GC-MS analysis of an authentic geraniol standard for retention time comparison. (**D**,**E**) Comparison of EI mass spectra between geraniol produced by (**D**) *N. benthamiana* expressing HMGR + P19 + ZmTPS27 (panel B, retention time 9.10 min) and (**E**) authentic geraniol standard (panel C, retention time 9.10 min).

Association mapping of the sesquiterpene alcohols resulted in a broad chromosome 1 mapping interval containing 2 candidate ZmTPS genes (Figure 2A–G; Table S3). Given the previous characterization of ZmTPS8 following transgenic expression in Arabidopsis [28], we first cloned (Table S4) and examined the uncharacterized B73 gene *ZmTPS27*. *Agrobacterium* mediated heterologous expression of *ZmTPS27* in *N. benthamiana* resulted in consistent production of the monoterpene geraniol, yet no identifiable sesquiterpenes (Figure 3A–D and Figure S2). Based on our current results and related studies [32], observable activity of the enzyme encoded by *ZmTPS27* is consistent with a monoterpene synthase instead of a sesquiterpene synthase.

### 2.4. Previously Undetected Maize Sesquiterpenoids Are Products of ZmTPS8

**Figure 4:**
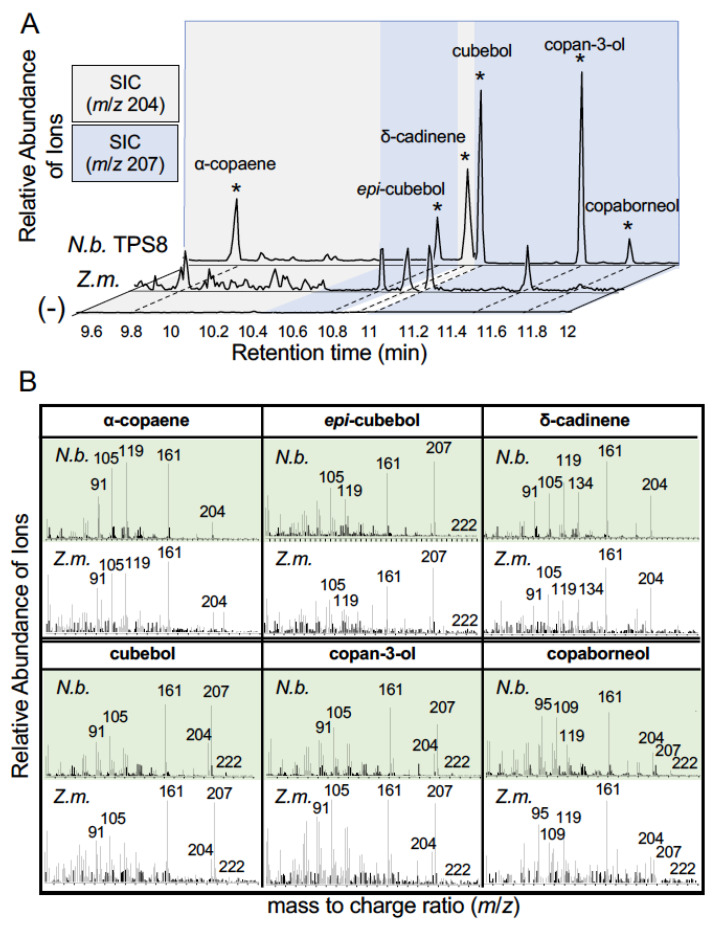
Heterologous expression of ZmTPS8 in *N. benthamiana* results in the production of α-copaene, *epi*-cubebol, δ-cadinene, cubebol, copan-3-ol and copaborneol. (**A**) GC/MS EIC following vapor-phase extraction of *N. benthamiana* leaf tissues expressing P19 + HMGR + ZmTPS8 (*N.b.* TPS8), fungal-elicited maize stem tissue from a B73 × M162W RIL (E0061) reference sample (*Z.m.*) and *N. benthamiana* expressing P19 + HMGR alone (-). Chromatograms were individually scaled. To highlight target analytes, the EIC retention time windows are subdivided into 4 segments alternating from *m*/*z* = 204 (grey) and *m*/*z* = 207 (blue) for visualization of endogenous ZmTPS8 products. (**B**) EI mass spectra for key analytes labeled in panel A. EI mass spectra highlighted in green are from *N. benthamiana* leaf tissues transiently expressing P19 + HMGR + ZmTPS8 while EI mass spectra in white are from the reference B73 × M162W RIL (E0061) fungal-elicited stem sample.

Previous research demonstrated that transgenic expression of *ZmTPS8* in *Arabidopsis thaliana* yielded production of α-copaene, (*E*)-β-caryophyllene, germacrene D and δ-cadinene resulting in classification of the enzyme as an α-copaene synthase (UniProtKB; Q29VN3) [28]. To investigate ZmTPS8 as the candidate enzyme responsible for the maize oxygenated sesquiterpenoids (Figure 1E and Figure 2A–F), we cloned the B73 *ZmTPS8* (Table S4) and heterologously expressed the enzyme in *N. benthamiana*. Metabolic profiling of tissue extracts demonstrated the production of α-copaene and δ-cadinene as expected but also contained four sesquiterpene alcohols consistent with *epi*-cubebol, cubebol, copan-3-ol and copaborneol (Figure 4A,B). Chromatographic retention times and EI spectra matched endogenous analytes detected in maize (Figure 4A,B) that likewise underlay the association mapping results (Figure 2). Unlike the production of (*E*)-β-caryophyllene and germacrene D previously observed in Arabidopsis expressing ZmTPS8 [28], these hydrocarbon olefins displayed little product accumulation (Figure 4A,B and Figure S2). Applied tissue extraction protocols using acidic 1-propanol or methanol resulted in further products derived from cubebol solvolysis such as 1-propanol and methanol derived adducts (Figure S3) [33]. An extension of this observation is that levels of cubebol directly detected likely result in under estimates of concentrations present in native tissues.

### 2.5. ZmCYP71Z19 Oxidizes a ZmTPS8 Product to Produce an Unknown Sesquiterpene Acid Which Exists Endogenously in Fungal-Elicited Maize Tissues

**Figure 5:**
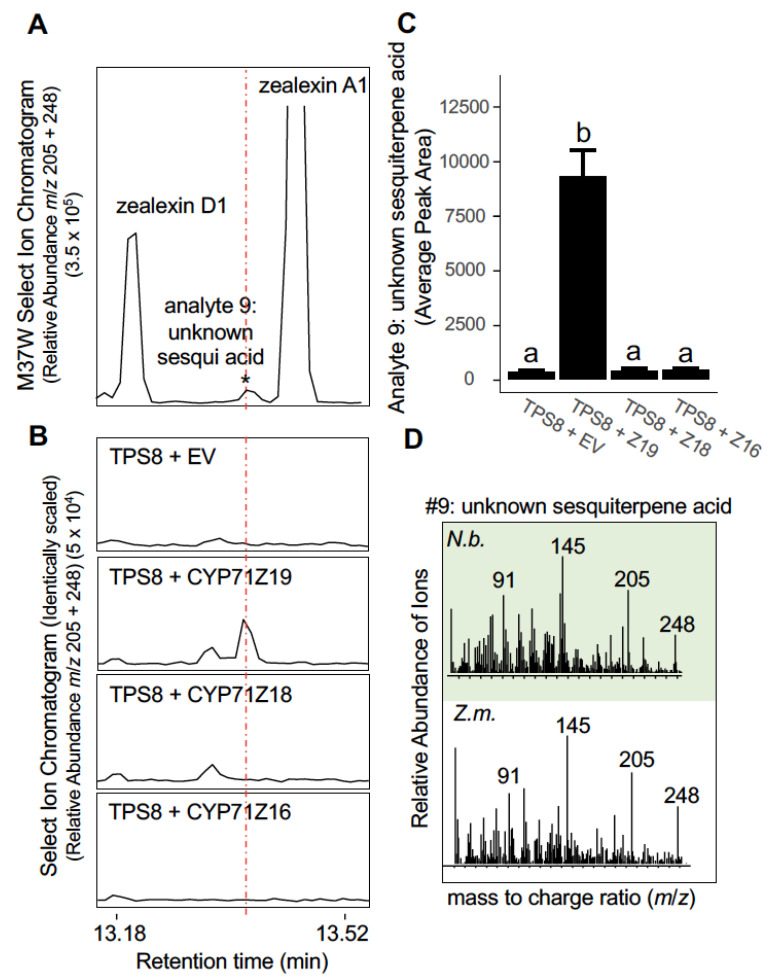
Combined heterologous expression of ZmTPS8 and ZmCYP71Z19 in *N. benthamiana* selectively results in the production of analyte 9, an unknown sesquiterpene acid. (**A**) GC/MS analyses of maize inbred line M37W as a reference sample (stem tissues 3 days after wounding and fungal elicitation) using an EIC (*m*/*z* 205 + 248) to highlight known sesquiterpene acids analyzed as methyl ester derivatives (zealexin D1 and zealexin A1). The dotted line represents retention time (RT) 13.37 min, 0.05 min before zealexin A1 as diagnostic reference RT for analyte #9. (**B**) Representative GC/MS SIC (*m*/*z* = 205 + 248) of *N. benthamiana* leaf tissue expressing the empty vector (EV: which includes P19 + HMGR) plus ZmTPS8 in further combinations with established cytochrome P450s involved zealexin biosynthesis, specifically Zx5 (ZmCYP71Z19), Zx6 ZmCYP71Z18) and Zx7 (ZmCYP71Z16). ZmTPS8 + EV alone is included as a control for interpreting ZmCYP71Z activity. SIC are identically scaled, with a cutoff abundance of 5 × 10^4^. (**C**) Average (N = 4, + SEM) peak areas of analyte 9, the unknown sesquiterpene acid (*m*/*z* 248) from replicated heterologous expression trials associated with panel B. Different letters (a, b) represent significant differences (one-way ANOVA followed by Tukey’s test corrections for multiple comparisons; *p* < 0.05). (**D**) Comparative EI mass spectra (highlighted in green) from *N. benthamiana* leaf tissues transiently expressing P19 + HMGR + TPS8 + ZmCYP71Z19 in panel B and M37W-derived EI mass spectra for analyte 9 (highlighted in white) from panel A.

ZmCYP71Z19, also termed Zx5 with a role in zealexin biosynthesis, is a fungal-regulated P450 at the transcriptomic and proteomic level with high substrate promiscuity yet measurable specificity for sesquiterpene substrates, when compared to ZmCYP71Z16 and ZmCYP71Z18 [17]. Based on a predicted ability to function in multiple sesquiterpenoid defense pathways in parallel, we tested ZmTPS8 in combination with ZmCYP71Z19, Z18 and Z16 via heterologous enzyme co-expression in *N. benthamiana*. ZmTPS8 co-expression with ZmCYP71Z19 resulted in the production of an unknown sesquiterpene acid (Figure 5 and Figure S4) displaying an identical GC retention time and EI spectra as analyte 9 (Figure 1B,E and Figure 5). In contrast to ZmCYP71Z19, heterologous co-expression of ZmTPS8 with ZmCYP71Z18 or ZmCYP71Z16 was insufficient for the production of the ZmTPS8-derived sesquiterpene acid (analyte 9) (Figure 5B,C and Figure S4). Given the multiproduct nature of ZmTPS8 [28], results from the current effort (Figure 4) and the catalytic ability of ZmCYP71Z19 to oxidize different sites within a single sesquiterpenoid hydrocarbon [18], we are currently unable to predict or establish the precise identity of the ZmTPS8-ZmCYP71Z19-derived sesquiterpene acid now demonstrated to occur in maize (Figure 5A–D).

### 2.6. Antifungal ZmTPS8 Products Are Linked to Complex Pathway Regulation Involving Both Wounding and Fungal Elicitation at Different Biosynthetic Steps

**Figure 6:**
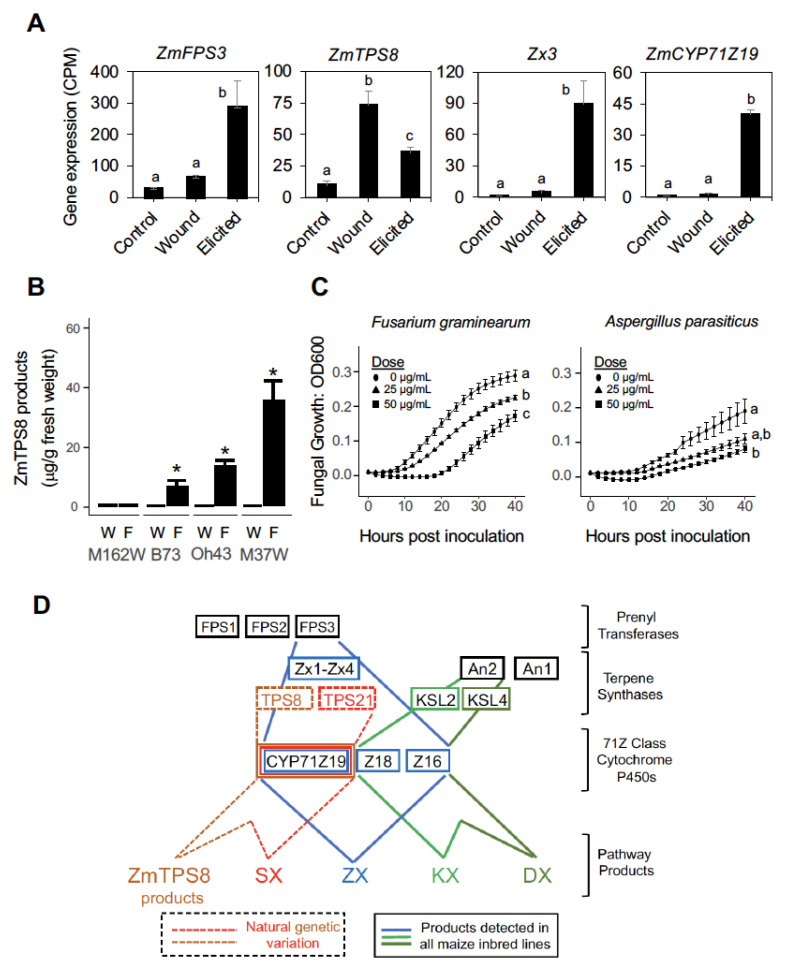
Genotype specific ZmTPS8-derived sesquiterpenoid antibiotics accumulate strongly following interactions between wounding and fungal elicitation to fuel larger defense-related biosynthetic networks. (**A**) Average (*n* = 3–4, SEM) gene expression (3′-RNA-seq) of *ZmFPS3*, *ZmTPS8*, *Zx3* and *ZmCYP71Z19* summarized as counts per million reads (CPM; B73 RefGen V4) in B73 maize stems that were either untreated (Control), damaged (Wound) or those damaged and elicited with heat-killed *F. venenatum* elicitor (Elicited). Samples were harvested 36 h later. Different letters (a, b, c) represent significant differences (one-way ANOVA followed by Tukey’s test corrections for multiple comparisons; *p* < 0.05). Wound and fungal-elicited treatments have been previously published [17]; however, representative untreated Control samples now exist here as newly analyzed, released and updated contributions to NCBI GEO (GSE138962) (see also Tables S5 and S6). (**B**) Average (*n* = 3, +SEM) quantification (mg g^−1^ fresh weight (FW)) of ZmTPS8 derived sesquiterpene alcohols (sum of *epi*-cubebol, cubebol, copan-3-ol and copaborneol) accumulating in replicated M162W, B73, Oh43 and M37W stem tissues 3 days after either wounding alone (W) or wounding plus fungal elicitation (F). Metabolite quantification estimates were derived from GC/MS EIC (*m*/*z* 207) peak areas and calculated based upon an external standard curve generated by spiking a set of known amounts of purified cubebol into separate wounded B73 stem tissue aliquots lacking observed metabolites. Within each inbred line, statistically significant differences were determined by pairwise Student’s t-test (*p* < 0.05). (**C**) Average (*n* = 8, ±SEM) fungal growth (relative to time zero) for maize pathogens *Fusarium graminearum* (**B**) and *Aspergillus parasiticus* (**C**) inoculated in minimal growth media containing cubebol at either 0 (●), 25 (▲) or 50 (■) μg mL/mL. Significance codes were assigned using one-way ANOVA and Tukey’s Honest Significant Differences Test at 40 h. (**D**) Proposed general pathway model for ZmTPS8 products (Brown) in context and co-occurrence with and interacting portions of the Zealexin (ZX; Blue), α/β-selinene (SX; Red), Kauralexin (KX; light green) and Dolabralexin (DX; dark green) pathways [3,17,18,26]. A list of gene abbreviations, B73 RefGen_V2/V4 gene IDs, protein IDs, and primary references are listed in Table S1. Transcriptomic results support the existence of a fungal-elicited *ZmFPS3* and heterologous expression assays support ZmCYP71Z19 in the production of analyte 9, a sesquiterpene acid that is the product of predominantly wound-activated transcript accumulation of *ZmTPS8*. Unlike activation of ZX, SX, KX and DX pathways, *ZmTPS8* expression has a significant wound-regulated component that appears to require fungal elicitation of further biosynthetic pathway genes (panel A) to yield detectable antibiotic products (panel B).

To consider the regulation of ZmTPS8 pathway products at the transcriptional level, we re-examined an existing 3′-RNA-seq study in the B73 inbred (NCBI GEO: GSE138962) [17] and further included unwounded control samples generated in the same experiment that had not been previously analyzed. The complete 36 h transcriptomic dataset of unwounded controls, wounded and fungal-elicited stems is now included in the current effort (Tables S5 and S6). Plant sesquiterpene synthases most commonly require FPP substrates supplied by prenyl transferase genes encoding FPP synthases (FPS). Maize encodes 3 distinct *ZmFPS* genes, namely, *ZmFPS1*, *ZmFPS2* and *ZmFPS3*. Systematic genetic mutant analyses of the *ZmFPS* family recently demonstrated that ZmFPS3 is specifically required for significant production of sesquiterpenoid antibiotics following fungal elicitation [17,34]. With significant co-expression, the role of ZmFPS3 in fungal regulated defenses is further supported at the proteomic level [17]. Consistent with established patterns, *ZmFPS3* and *Zx3* transcripts are strongly fungal-elicited and display no statistical differences between control and wounded tissues (Figure 6A,B). Unlike *Zx3*, *ZmTPS8* transcript accumulation displays the largest increase following wounding and is also elevated, yet to lower levels, with the further addition of fungal elicitors (Figure 6A). Contributing to zealexin and costic acid biosynthesis [17], *ZmCYP71Z19* transcripts are closely tied to fungal elicitation and track *ZmFPS3* and *Zx3* responses (Figure 6A,D).

To consider the connection more closely between wounding, fungal-elicitation and accumulation of ZmTPS8 products, we examined the M37W maize inbred previously displaying the greatest product accumulation (Figure 1D). To enable metabolite quantification, we created an external standard curve derived from HPLC purified cubebol spiked into control maize tissues. Samples were extracted using standard protocols which then accounted for partial losses in recovery due to the formation of cubebol solvolysis products (Figure S3). This effort confirmed that while wounding alone strongly promotes B73 *ZmTPS8* transcript accumulation (Figure 6A), ZmTPS8 products are non-detectable in diverse stem tissues following wounding alone (Figure 6B). Instead, ZmTPS8 products are only present in the combined wound + fungal elicitation treatments and maximally accumulate in M37W to approximately 40 μg g^−1^ FW (Figure 6B). Using purified cubebol, we then tested doses within this range, namely 25 and 50 μg mL^−1^ for antibiotic activity against *Fusarium graminearum* and *Aspergillus parasiticus* (Figure 6C). At the lowest dose tested, 25 μg mL^−1^ cubebol was sufficient to suppress growth of both fungal pathogens. Our results are consistent with ZmFPS3 driving the production of fungal-elicited sesquiterpenoid antibiotics [17,34], but extends the observation to ZmTPS8 which displays preferential wound-mediated transcript accumulation. We hypothesize that wound-regulated *ZmTPS8* encoded enzymes may be limited in FPP precursors that are supplied by the catalytic activity of fungal-regulated ZmFPS3 accumulation. ZmTPS8 pathway regulation appears to involve combinatorial interactions that are not identical to the more closely examined ZX and KX pathways (Table S6).

As a current working model (Figure 6D), previously established maize terpenoid antibiotics include kauralexins (KX), dolabralexins (DX), zealexins (ZX) and α/β-selinene derivatives (SX). ZmTPS8 products further occur within this larger interconnected metabolic network. Like KX, DX, ZX and SX precursors, ZmTPS8 products can interact with the catalytically promiscuous enzymes derived from the chromosome 5 CYP71Z gene cluster that act on diverse endogenous substrates (such as α/β-selinene, β-bisabolene, β-macrocarpene, dolabradiene) and display partially overlapping substrate specificity to generate diverse products. Like the ZmTPS21 α/β-selinene synthase [3], observed ZmTPS8 product formation is genetically variable and contributes to the cocktail of antibiotics generated by the chromosome 5 gene cluster encoding ZmCYP71Z19, Z18 and Z16 enzymes that drive a complex hourglass-shaped biosynthetic pathway (Figure 6D).

To finally consider if comparatively higher levels of observed ZmTPS8 products (Figure 1D) display predictive associations with genetically encoded ZmTPS8 amino acid (AA) sequences, we constructed a protein phylogeny that included the replicated inbred lines used in this study (Table S7, Figure S5). Inbred lines displaying detectable ZmTPS8 product accumulation, including B73 and the top 4 highest average lines, namely M37W, IL14H, B97 and Oh43 (Figure 1D), were found to form a distinct subclade within the 29 inbred lines analyzed (Figure S5). While precise mechanisms at the AA level driving this pattern have not been demonstrated, the underlying nucleotide sequences likely correspond with the ability to predict inbred lines capable of ZmTPS8 product accumulation. 

## 3. Discussion

Plants are protected from biotic attack in part by complex arrays of constitutive and inducible specialized metabolites that are commonly derived from fatty acid, amino acid, phenylpropanoid and isoprenoid pathways [35,36]. In maize, direct protection against herbivory is commonly driven by high levels of reactive nitrogen containing benzoxazinoids present in young plants [4,37,38]. For indirect defenses, maize relies on dynamic herbivore-elicited production of mono-, homo- and sesquiterpenes volatiles that collectively contribute to the attraction of natural enemies such as parasitoid wasps and entomopathogenic nematodes [10,13,14,19,39]. In contrast, fungal-elicitation results in strong suppression of early benzoxazinoid biosynthetic proteins and an activation of a subset of terpene synthase transcripts and proteins largely separate from those associated with herbivory [17,18]. Motivated by opportunities afforded by rich genetic diversity in maize [40] and expansive proteomic changes following fungal elicitation [17], we used metabolic profiling to further uncover additional oxygenated terpenoids likely to contribute to complex antibiotic cocktails present in microbially challenged tissues. Combining association mapping and hypothesis testing using heterologous protein expression assays, we connected a series of uncommonly observed maize sesquiterpenoids to ZmTPS8. Newly detected sesquiterpene alcohols were consistent with *epi*-cubebol, cubebol, copan-3-ol and copaborneol. As a further endogenous maize metabolite, an unknown ZmTPS8-derived acid was biosynthetically confirmed as a modular product of ZmCYP71Z19 but not related ZmCYP71Z18 or ZmCYP71Z16 enzymes. A specific ZmTPS8 product, namely cubebol, was demonstrated to have growth inhibitory activity against maize fungal pathogens at concentrations of 25 μg mL^−1^. Curiously, ZmTPS8 product accumulation corresponded more with fungal elicited *ZmFPS3* levels than the predominantly wound-inducible *ZmTPS8* transcript levels (Figure 6A). Thus, in select inbred lines, ZmTPS8 products partially track fungal-elicited zealexin accumulation, but this occurs through different patterns of transcript regulation suggesting precursor limitations under conditions involving wounding alone. Detectable ZmTPS8 products in challenged tissues partially resemble the ZmTPS21 products, such as α/β-selinene derivatives [3], in significant ways. Firstly, ZmCYP71Z19 selectively oxidizes pathway products further [17] and, secondly, common genetic variation exists which is potentially useful for breeding purposes.

Early investigations of dynamically regulated specialized metabolites in maize stems following European corn borer (*Ostrinia nubilalis*) herbivory revealed unexpected mixtures of oxygenated sesquiterpenoids and diterpenoids, termed zealexins (ZX) and kauralexins (KX), respectively [41,42]. However, compared to herbivory, fungal elicitation resulted in an order of magnitude greater than KX and ZX accumulation in stems consistent with a predominant role in microbial interactions. Subsequent GC/MS-based metabolic profiling efforts in diverse maize germplasm challenged with fungal elicitors and natural field conditions further revealed α/β-selinene derivatives (SX) such as β-costic acid [3] and dolabradiene derivatives, termed dolabralexins (DX), such as epoxydolabranol [26]. While over 30 fungal-elicited maize terpenoid antibiotics have been identified, further related metabolites contributing to the complex blend remain unresolved. Our current analyses revealed a subset of inbred lines capable of accumulating rarely encountered sesquiterpene alcohols consistent with *epi*-cubebol, cubebol, copan-3-ol and copaborneol (Figure 1A–E). Unlike KX, DX and ZX detectable in all maize lines, this set of sesquiterpene alcohols was present in less than half of the examined germplasm (Figure 1D) and conceptually resembles SX defenses [3].

Qualitative variation in the model cubebane sesquiterpene, cubebol, prompted assessment of the B73 × M162W RIL mapping subpopulation [29]. Association mapping with cubebol, *epi*-cubebol, copan-3-ol and copaborneol displayed similar statistically associated SNPs on chromosome 1 (Figure 2A–D). Furthermore, a single unknown sesquiterpene acid (analyte 9) mapped to the same locus on chromosome 1 in both the B73 × M162W RILs and the Goodman association mapping population (Figure 2E,F). Two *ZmTPS* genes spanned this broad mapping interval, namely, *ZmTPS27* and *ZmTPS8* (Figure 2G; Table S3). Consistent with linked biosynthesis, metabolite-based Mutual Rank analyses supported tightly tracked co-abundance patterns (Figure 2H). While association mapping approaches have been successfully used for late stage *ZX* and *KX* biosynthetic genes, they have not provided further support for the highly conserved or duplicated underlying *ZmTPS* genes [17,18].

Based on the existing maize literature [7], it was unclear if target analytes such as cubebol were derived from ZmTPS27 or ZmTPS8. We first examined the previously uncharacterized gene *ZmTPS27* for cloning and expression in *N. benthamiana*. Heterologous enzyme expression and analyses of product profiles yielded only geraniol, a simple linear monoterpene alcohol (Figure 3). To date, native enzymes capable of geraniol biosynthesis in maize have not been specifically targeted for discovery; however, early characterization of ZmTPS1 (GRMZM2G049538) demonstrated an ability to utilize GPP as a substrate to yield a mixture of linalool and geraniol [43]. Geraniol acetate is a common component of maize leaf volatile emissions following insect oral secretion elicitation and displays distinct regulation pattens under different abiotic stress conditions [44]. Phylogenetic of analyses *ZmTPS27* (AC205502.4_FG004) place it in the TPS-a subfamily clade V [16] which include proven plastid localized examples of monoterpene synthases in Poaceous grain crops [45]. While we have not yet detected significant endogenous levels of geraniol or derivatives in fungal-elicited maize tissues it is possible geraniol is produced as a transient intermediate or contributes to herbivore-elicited geranyl acetate emission in leaves [44]. In support of these ideas, previous studies demonstrated that transgenic expression of a geraniol synthase from *Lippia dulcis* in maize resulted in geranyl acetate and the predominant accumulation of a geraniol glycosides, geranic acid glycosides and hydroxyl-geranic acid glycosides [46]. Collectively, our efforts to characterize *ZmTPS27* present in the metabolite mapping interval (Figure 2G) are inconsistent with a sesquiterpene synthase.

ZmTPS8 has been characterized as a sesquiterpene synthase through transgenic expression in Arabidopsis resulting in predominant volatile products that include α-copaene, (*E*)-β-caryophyllene, germacrene D and δ-cadinene [28]. *Ostrinia furnacalis* herbivory in whorl tissues of the maize genotype Jingke968 strongly enhanced accumulation of *ZmTPS8* transcripts and production of germacrene D, α-copaene and α-cubebene [47] further suggesting endogenous associations. In fungal-elicited B73 maize stem tissues, α-copaene (Figure 1A) along with cubebol and related sesquiterpene alcohols, exists at low yet detectable levels. Detailed analyses of *Medicago truncatula* TPS5 (MtTPS5) demonstrates that germacrene D can undergo further carbocation rearrangements to generate diverse products which include α-copaene, β-copaene, cubebol, copan-3-ol, α-cubebene, β-cubebene, δ-cadinene and many others [48]. Based on these foundational studies and the exclusion of ZmTPS27, ZmTPS8 remained as the main candidate responsible for the sesquiterpene alcohols detected in fungal-elicited stems. Direct comparison of GC/MS retention times, EI spectra of analytes derived from maize stems and *N. benthamiana* leaves following the heterologous expression of ZmTPS8 resulted in matching identified products (Figure 4). We speculate that higher boiling points and lower volatility associated with the ZmTPS8-derived sesquiterpene alcohols reported on in the current effort may be responsible for reduced detection in earlier studies focused on the more volatile hydrocarbon olefins [28]. Based upon our replicated metabolite analyses in genome sequenced inbred lines and the phylogenic analysis of corresponding ZmTPS8 amino acid sequences (Table S7), B73 and inbred lines with the highest average ZmTPS8 product accumulation, namely, M37W, Il14H, B97, Oh43 (Figure 1D) are all members of the same phylogenic subclade (Figure S4). The correspondence between high levels of product accumulation and a specific ZmTPS8 subclade supports the existence of predictability in this genetically variable biochemical phenotype.

Maize terpenoid antibiotics are increasingly appreciated to be derived from diverse sesquiterpene and diterpene hydrocarbon olefins that are oxygenated by an expressed gene cluster of 3 closely related ZmCYP71Z proteins present on chromosome 5 [17,18,26,49]. Relevant endogenous maize substrates support specific members of the ZmCYP71Z protein family in the oxidization of β-selinene, β-bisabolene, β-macrocarpene, *ent*-kaurene, *ent*-isokaurene and dolabradiene [17,18,26]. Collective results strongly suggest that an even wider range of both native endogenous and experimentally tested substrates can be catalytically oxidized. To better understand why an unknown sesquiterpene acid (analyte 9) also displayed association mapping to the ZmTPS8 interval (Figure 1B,E and Figure 2E,F), we tested pairs of heterologously expressed protein combinations of ZmTPS8 individually with ZmCYP71Z19, ZmCYP71Z18 and ZmCYP71Z16. Our results support that the combined activity of ZmTPS8 and ZmCYP71Z19 produces a biochemical with identical EI spectra and retention time matching the endogenous sesquiterpene acid (analyte 9) (Figure 5). While low levels of product accumulation in maize and *N. benthamiana* made large scale isolation and identification efforts impractical, our results support ZmTPS8 as a further endogenous contributor to the complex and genetically variable blend of acidic maize terpenoid antibiotics (Figure 6D). Previous gene evolution estimates are consistent with a single related *CYP71Z19* gene that was present in the progenitor to both sorghum and maize, and that the *CYP71Z* gene subsequently duplicated and diverged in maize [17]. In our previous and current efforts, empirical data support ZmCYP71Z19 as a comparatively selective enzyme for sesquiterpene substrates (ZmTPS8 products, α/β-selinene, β-bisabolene, β-macrocarpene) which likewise displays only weak activity on diterpene substrates such *ent*-isokaurene [17]. 

A detailed study of sesquiterpenes in B73 [50] demonstrated that ZmTPS8 products [28] such as α-copaene, δ-cadinene and germacrene D are present in a defined co-regulated module detectable in all B73 seedling tissues. In mature plants, ZmTPS8 products are found in husk tissues but not mature leaves, tassels or silks [50]. B73 ZmTPS8 product profiles exhibited unique pathway regulation and enabled accurate group classification prior to understanding the underlying TPS responsible [28,50]. In the current effort, we focused on 35 to 40-day old stem tissues challenged with fungal-elicitation and tracked biochemical and transcriptomic responses. Unlike the representative β-macrocarpene synthase encoding transcript *Zx3*, strongly regulated by fungal elicitation, *ZmTPS8* is predominantly wound-inducible (Figure 6A). Despite this observation and associated parsimonious predictions, stem wounding alone does not result in the accumulation of detectable ZmTPS8 products (Figure 6B). Based on comprehensive proteomic patterns and defined mutant analyses, the ZmFPS most significantly responsible for providing increased FPP substrates to fungal-elicited sesquiterpenoid defenses is ZmFPS3 [17,34]. Our detection of significant ZmTPS8 products in stems is consistent with an important combination of both wound-induced *ZmTPS8* transcript accumulation and fungal-elicited *ZmFPS3* transcript accumulation (Figure 6A). Taken as a whole, the regulatory patterns of ZmTPS8 products and pathway transcripts contrasts simple and strong regulatory patterns found in much of the ZX pathway [17]. The current example more closely supports intersecting pathway interactions between wound-regulated *ZmTPS8*, previously established as either a constitutive [50] or herbivory-elicited pathway [47], and fungal-elicited processes such as the strong up-regulation of *ZmFPS3* and *ZmCYP71Z19*. It is well appreciated that combinations plant of TPS and CYP enzymes enable dynamic modular combinations capable of accepting diverse precursors to generate further decorated products [51]. Our results are consistent with an endogenous example where fungal-regulated enzymes both up-stream (ZmFPS3) and downstream (ZmCYP71Z19) of ZmTPS8 can interact with a functional TPS neither previously nor necessarily associated with microbial-elicited defenses. We speculate that examples of complex combinatorial pathway interactions exist as important biochemical phenotypes which could be further acted upon by positive selection pressures to generate additive layers of biochemical immunity over evolutionary time. Independent association studies have linked *ZmTPS8* to phenotype classes which include nutrition and disease but also ear traits, flowering time, leaf morphology and tassel architecture [52]. In specific maize germplasm, ZmTPS8 products exist as additive components of a complex terpenoid antibiotic cocktail present following elicitation that are not readily predicted by transcriptomic co-expressions patterns alone. Our identification of a specific subclade of comparatively high ZmTPS8 product accumulating lines (Figure S5) provides new information for plant breeding approaches that seek to include diverse functional layers of biochemical immunity.

## 4. Materials and Methods

### 4.1. Plant and Fungal Materials

Seeds of the Goodman diversity panel and the nested association mapping (NAM) B73 × M162W RIL subpopulation for association analyses were kindly provided by Dr. Georg Jander (Boyce Thompson Institute, Ithaca, NY, USA) and by Dr. Peter Balint-Kurti (USDA-ARS, Raleigh, NC, USA), respectively (Table S2). Seeds of the NAM parental lines [29] were obtained from the Maize Genetic COOP Stock Center, Urbana, IL, USA. Unless otherwise mentioned, maize inbreds used for replicated elicitation experiments were germinated in MetroMix 200 (Sun Gro Horticulture Distribution, Inc., Bailey, CO, USA) supplemented with 14-14-14 Osmocote (Scotts Miracle-Gro, Marysville, OH, USA) and grown in a greenhouse for 35 days prior to initiation of experiments. Fungal stock cultures of *Fusarium graminearum* (NRRL stock no. 31084) and *Aspergillus parasiticus* (nor-1) were grown on V8 agar for 10 days before the quantification and use of spores [42]. Heat-killed *Fusarium venenatum* (strain PTA-2684) hyphae was commercially obtained (Monde Nissin Corporation Co., Santa Rosa, Laguna, Philippines) and used as a non-infectious elicitor lacking known mycotoxins.

### 4.2. Maize Stem Tissues Used for Metabolite-Lead Association Analyses

Association mapping using biochemical traits described in this study follow from previously described experiments [18]. Briefly, the B73 × M162W RIL subpopulation [29] and Goodman diversity panel [53] were planted at the University of California San Diego (UCSD) Biology Field Station La Jolla, CA, USA in the summer of 2016. To avoid plant response variation due to the action of fungal effectors on diverse lines, all replicated NAM parent experiments and mapping experiments used slit stem treatments coupled with the addition of heat-killed *F. venenatum* hyphae. Here, crude *F. venenatum* was homogenized in a Waring blender in the presence of additional water at 20% (*w*/*w*) to create a smooth paste. Approximately 500 μL of fungal elicitor was introduced into the longitudinally slit apical meristem followed by sealing the site with packing tape to minimize desiccation of the treated stem tissues. Elicited maize stems were harvested five days later, frozen in liquid N_2_, ground to a powder and stored at −80 °C for analyses. For individual experiments, details relating to biological replications and harvest time points are noted in the figures and captions.

### 4.3. Identification and Analyses of Plant Metabolites

Sample preparation relied on vapor phase extraction (VPE) to remove high molecular weight analytes incompatible with gas chromatography (GC) [54]. Using a modified VPE procedure, 50 mg sample aliquots finely ground in liquid N_2_ were extracted with 300 μL 1-propanol (0.1% HCl) followed by the addition of 1 mL hexane in a 4 mL glass vial. The resulting organic phase was transferred to a new vail and derivatized using clean trimethylsilyl diazomethane. Following 20 min for derivatization, all remaining liquids were carefully dried under a N_2_ stream with precautions taken to avoid over-drying which could result in a loss of volatile plant-derived analytes. Key modifications from previous sample preparation [54] now avoid the use of plasticware at all steps prior to VPE to minimize sources of phthalate contamination found to permanently degrade chromatographic behavior of semipolar analytes. Final analytical samples were eluted from the VPE traps using 150 μL of 1:1 hexane: ethyl acetate. GC/MS analyses were conducted using an Agilent 6890 series gas chromatograph joined to an Agilent 5973 mass selective detector (mass temperature, 150 °C; interface temperature, 250 °C; electron energy, 70 eV; source temperature, 230 °C). A DB-35 MS column (Agilent; 30 m × 250 μm × 0.25 μm film) was used for gas chromatography. Samples were introduced using a splitless injection into a 200 °C inlet with an initial oven temperature of 45 °C. The temperature was held for 2.25 min, then increased to 300 °C with a gradient of 20 °C min^−1^ and held at 300 °C for 5 min. A solvent delay of 4.5 min was selected to prevent ethyl acetate present in the sample from damaging the EI-filament. GC/MS-based estimates of ZmTPS8 product levels were based upon an external standard curve of HPLC purified cubebol spiked into unwounded control maize stem tissue samples lacking ZmTPS8 products. Agilent Mass Hunter Qualitative and Quantitative Analysis software, alongside Agilent ChemStation qualitative programs, were used to generate and analyze the GC/MS generated chromatograms and spectra. Replicated experiments were summarized with peak areas captured by MassHunter Qualitative Navigator B.08.00, and MS Quantitative Analysis B.08.00, quantified in Excel, and statistically evaluated in JMP. MassHunter-based peak selection methods were carefully created to take into account retention time shifts. As needed, automated peak integrations were substituted for manual integrations of target compounds in select samples. GC/MS analyses of *N. benthamiana* leaves with different combinations of heterologously expressed proteins follows directly from the analyses of maize tissues.

Identification of methyl ester derivatives of established ZX and KX products were based on relative retention times and published reference spectra [17,18,41,42]. Where possible, ZmTPS27 and ZmTPS8 product identification was conducted using authentic standards for geraniol, α-copaene, germacrene D, δ-cadinene, *epi*-cubebol (CAS# 38230-60-3), cubebol (CAS# 23445-02-5) and matching EI reference spectra in the Robert P. Adams Essential Oil MS library [55] and the National Institute of Standards and Technology (NIST) library. *Piper cubeba* essential oil was used as a source of reference standards for *epi*-cubebol and cubebol [56]. Less commonly encountered sesquiterpene alcohols copan-3-ol (CAS# 133647-18-4) copaborneol (CAS# 21966-93-8) were identified based on close EI spectra matches from defined MtTPS5 products involving protonation of the neutral intermediate germacrene D [30]. Diverse established MtTPS5 products include germacrene D, α-copaene, δ-cadinene; cubebol, copan-3-ol, copaborneol; and additional related products [30].

### 4.4. Maize Forward Genetics Studies Using Metabolite Association Analyses

A list of RILs and inbred lines used for association mapping in this study is provided (Table S2). Prior to statistical analyses, metabolite data for the unknown sesquiterpenoid analytes (3, 4, 5, 6, 9, S1, S2) were transformed using manually assigned accumulation codes as follows: 0; no analyte detected, 1; weakly detectable spectra with low signal-to-noise ratio thresholds, 2; clear spectra present with an unambiguous chromatographic peak, 3; clean spectra present with clear chromatographic peak constituting a top 10% (within class) analyte abundance level across the mapping population. Genetic marker data for the B73 × M162W RIL subpopulation (July 2012 All Zea GBS final build) were downloaded from www.panzea.org, accessed on 15 January 2018. Differential population structure and familial relatedness issues are uncommon as significant features in biparental RIL populations, thus the general linear model (GLM) was used for simple association analyses [3]. GWAS analyses utilized the B73 RefGen_v2 HapMap consisting of 246,477 SNPs as described [57]. GWAS was conducted using log2 transformed analyte abundances as traits and the unified mixed linear model in TASSEL 5.0. SNPs with less than 20% missing genotype data and minor allele frequencies > 5% from both an Illumina 50K array and a genotyping by sequencing (GBS) strategy were employed [18,57]. Analyses were performed in TASSEL 5.0 [58], and Manhattan plots were constructed in the R package qqman.

### 4.5. Transient Heterologous Co-Expression Assays in N. benthamiana

B73 cDNA RACE library construction and general cloning strategies for the full-length open reading frames of *ZmTPS27* and *ZmTPS8* directly followed from previously described methods [18] using cloning and sequencing primers as listed (Table S4). For transient expression in *N. benthamiana*, *Agrobacterium tumefaciens* strain GV3101 cells were transformed via electroporation with pLIFE33 constructs carrying individual target genes and pEarleyGate100 plant expression vectors containing full-length open reading frames (Table S4). Transformed cells were grown at 28 °C for 24 h in Luria Bertani media containing 50 µg mL^−1^ kanamycin, rifampicin and 30 µg mL^−1^ gentamycin. Cells were harvested and resuspended at a final OD_600_ of 0.8 in 10mM MES buffer and 10 mM MgCl_2_. All assays further utilized P19 and co-expression of the coding sequence for truncated cytosolic *Euphorbia lathyris* 3-hydroxy-3-methylglutaryl-coenzyme A reductase (HMGR; ElHMGR^159–582^, JQ694150.1) combined at equal concentrations to increase sesquiterpene product accumulation [59]. The resulting cell culture solutions were infiltrated via blunt syringe into the leaves of 35 day-old *N. benthamiana plants*. Plants were grown for 5 days under normal light conditions, after which infected tissues were frozen in liquid N_2_ and ground into a fine powder and stored at −80 °C for further analysis.

### 4.6. 3′-RNA-Seq Analyses of Control, Wounded and Fungal-Elicited B73 Stems

To consider B73 transcripts present in (A) unwounded control tissues compared to those (B) wounded, or (C) wounded with additional fungal elicitors for 36 h, we completed the examination of a partially analyzed and partially submitted public dataset of 3′-RNA-seq data at NCBI Gene Expression Omnibus (GEO; GSE138962). Sample preparation for the 3′ RNA-seq library construction and sequencing has been previously described in detail [17]. New additions to the current effort include the deposition and analyses of unwounded control (*n* = 4) stem samples generated and sequenced previously at the same time but were neither analyzed nor submitted as they were unessential to assess the contribution of fungal elicitation to wounding. We have now uploaded submission of the parallel unwounded control tissues to GEO (GSE138962) and supply the updated combined analyses of B73_V4 transcript levels normalized to counts per million (CPM) (Tables S5 and S6). Raw reads were filtered using FASTP (v0.23.2, default parameters) [60] and aligned to the maize B73 RefGen_V4 reference genome obtained from Phytozome [61] using HISAT2 (v2.2.0, -max-intronlen 6000) [62]. The alignment files were sorted and indexed using SAMBAMBA (v0.8.2, default parameters) [63] prior to using featureCounts package from subread (v2.0.1, -t exon, CDS) [64] for generating read counts. edgeR (v3.40.0) [65] was used to analyze raw read counts, including data normalization and manual outlier removal of a single sample (wound stem#1) based on multidimensional scaling plot (plotMDS), statistical analyses of between-group fold-changes (exactTest) and computing CPM.

### 4.7. Construction of the ZmTPS8 Phylogenetic Tree among Diverse Inbred Lines

Protein sequences of related ZmTPS8 gene models across different maize inbreds were obtained through MaizeGDB [66] (Table S7). The protein sequences were first aligned using famsa (v1.6.2) [67] and clipped using ClipKIT (v1.1.5) [68]. Then, IQ-TREE was used to automatically select the best phylogenetic model and to generate the final phylogenetic tree (v2.1.4, default parameters and 1000 bootstrap replications) [69,70]. The phylogenetic tree was visualized and annotated with FigTree (http://tree.bio.ed.ac.uk/software/figtree/, accessed on 5 September 2022.) (Figure S5).

### 4.8. Purification of Cubebol from Piper Cubeba Essential Oil

Cubebol was purified from *Piper cubeba* essential oil (Silky Scents, https://www.silkyscents.com/, accessed on 5 March 2020) using flash chromatography followed by high-performance liquid chromatography (HPLC). Five mL of commercial *P. cubeba* oil was first separated by preparative flash chromatography (CombiFlash^®^Rf, Teledyne ISCO, Inc., Lincoln, NE, USA) on a 40 g Silica (RediSepRF Gold) column. The mobile phase consisted of solvent A (hexane) and solvent B (ethyl acetate) with a continuous gradient of A starting at 100% to B (90%) from 5 min to 55 min using a flow rate of 40 mL min^−1^. Aliquots from individual 1 min fractions were screened by GC/MS for the presence of significant cubebol enrichment. Final HPLC purification utilized a Zorbax RX-silica (250 × 4.6 mm, 5 μm; Agilent) column with similar mobile phase A (hexane (100%) and B (ethyl acetate) using a linear gradient of mobile phase A starting at 0 min and reaching 90% mobile phase B at 40 min. One minute fractions were gently dried under a N_2_ stream, visually accessed for observable oil and analyzed by GC/MS for purity. Fractions derived from repeated injections and containing pure cubebol (Figure S5) were selected for downstream bioassays and tissue quantification efforts using standard addition experiments as an external standard curve.

### 4.9. In Vitro Antifungal Activity Assays with Cubebol

In vitro antifungal assays using HPLC purified cubebol from *Piper cubeba* essential oil were performed using the Clinical and Laboratory Standards Institute M38-A2 guidelines as detailed [41]. In brief, a 96-well microtiter plate-based method using a Synergy4 (BioTek Instruments, Winooski, Vermont, USA) reader was used to monitor fungal growth at 30 °C in broth medium through periodic measurements of changes in optimal density (OD_600_ nm) for 48 h. Each well contained 200 μL of initial fungal inoculum (2.5 × 10^4^ conidia mL^−1^; of *A. parasiticus* nor-1 and *F. graminearum*) with 1 μL of either ethanol or ethanol containing 5 or 10 μg cubebol to achieve final concentrations of 0, 25 and 50 ug mL^−1^ of the model ZmTPS8 derived antibiotic.

### 4.10. Statistics and Mutual Rank Analyses

Statistical analyses were conducted using JMP Pro v.16.0 (SAS Institute, Cary, NC, USA). One-way ANOVA were performed to evaluate statistical differences. Tukey tests were used to correct for multiple comparisons between control and treatment groups. Student’s unpaired two-tailed t-tests were conducted for simple pairwise comparisons. *p* < 0.05 were considered to be statistically significant. Analyses of metabolite peak area co-abundance within the B73 × M162W RIL subpopulation was examined using calculations of Mutual Rank scores using the public RStudio Graphical User Interface program MutRank as described [31].

## Data Availability

Newly submitted 3′-RNA-seq data for unwounded B73 stem tissue can be located at NCBI Gene Expression Omnibus (GEO; GSE138962).

## Supplementary Materials

The following supporting information can be downloaded at: https://www.mdpi.com/article/10.3390/plants12051111/s1, Figure S1: *Piper cubeba* essential oil contains cubebol and *epi*-cubebol in concentrations suitable for purification; Figure S2: Product profiles of *N. benthamiana* tissues following the heterologous expression of chromosome 1 mapping locus ZmTPS genes support the role of ZmTPS8 proteins underlying rarely detected maize terpenoids; Figure S3: Artifact formation of oxygenated sesquiterpenoid adducts through the acid solvolysis of ZmTPS8 products; Figure S4: Production of analyte 9, an unknown sesquiterpene acid across individual *N. benthamiana* plants expressing ZmTPS8 specifically with ZmCYP71Z19; Figure S5: ZmTPS8 amino acid sequences from B73 and the four inbred lines with the highest average ZmTPS8 product accumulation form a distinct subclade; Table S1: Genome and gene database identifiers for all annotated maize farnesyl diphosphate synthases (FPS), terpene synthases and select cytochrome P450 (CYP) enzymes; Table S2: Mapping lines used in this study; Table S3: Genes present in the chromosome 1 locus identified; Table S4: Primer sequences and sequence-validated gene coding sequences used in this study; Table S5: Heat-killed *Fusarium*-induced gene expression in B73 maize stems at 36 h; Table S6: Select B73 V4 transcripts of interest from Table S5 encoding biosynthetic enzymes relevant to maize terpenoid pathways with either established or potential roles in antifungal defenses and growth hormone production; Table S7: Maize inbred lines, predicted *ZmTPS8* gene models and encoded amino acid sequences used to construct the ZmTPS8 protein phylogeny. Refs. [71,72,73,74,75,76,77,78,79] can be found in Supplementary Materials.
