# Supplementary material for: Maize Terpene Synthase 8 (ZmTPS8) Contributes to a Complex Blend of Fungal-Elicited Antibiotics
Source: Plants (Basel). 2023 Mar 1;12(5):1111. doi: 10.3390/plants12051111 (PMC10005556; doi:10.3390/plants12051111)
Supplement: Supplementary file 1 [file plants-12-01111-s001.zip › Table_S_Fig_S_ZIP/230208_ZmTPS8_Figure_S1_5.pdf]

Figure S1

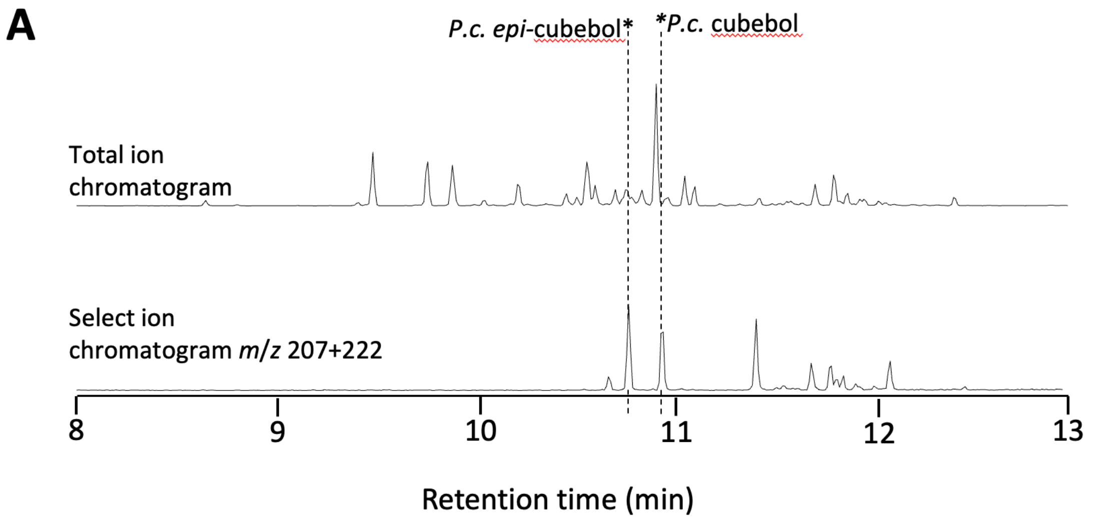

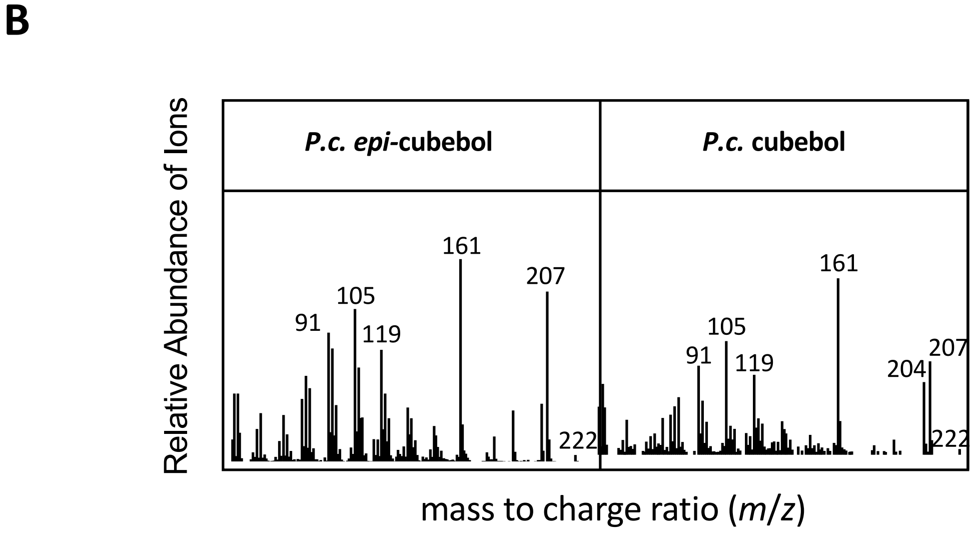

Figure S1: *Piper cubeba* essential oil contains cubebol and *epi*-cubebol in concentrations suitable for purification. (A) GC-MS TIC (top) and EIC (bottom, *m*/*z* 207 + 222) chromatographs of *Piper cubeba* essential oil obtained from a commercial source. Chromatographs were individually scaled for visual clarity, to highlight abundant peaks. The two first highly abundant alcohols by peak area are labeled. (B) EI mass spectra for the first 2 abundant alcohols labeled in A.

Figure S2

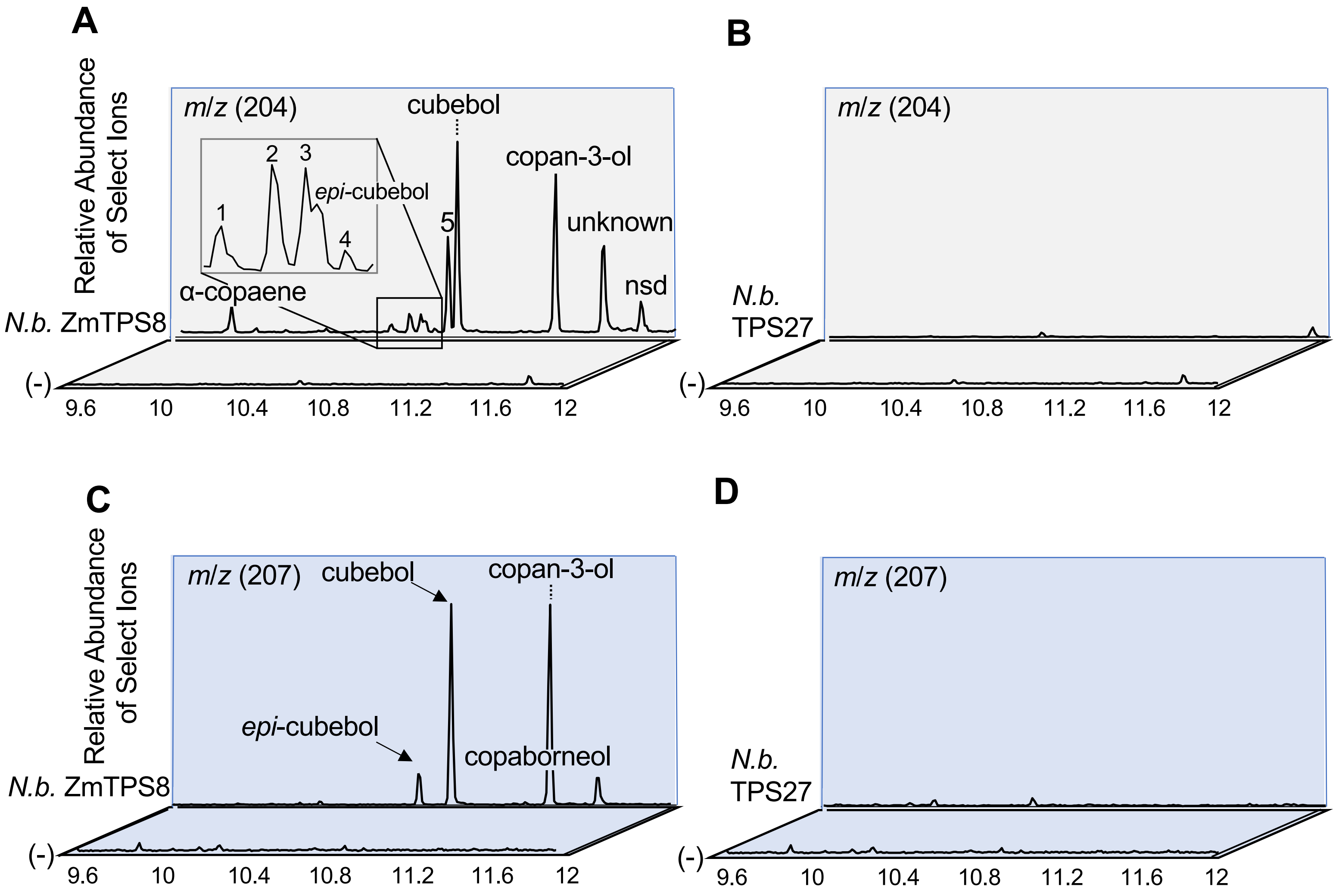

Figure S2: Product profiles of *N. benthamiana* tissues following the heterologous expression of chromosome 1 mapping locus *ZmTPS* genes support the role of ZmTPS8 proteins underlying rarely detected maize terpenoids. GC-MS EIC (hydrocarbon olefin parent ion; *m/z* 204) chromatographs of (A) ZmTPS8 and (B) ZmTPS27 and EIC (re-occurring sesquiterpene alcohol fragment ion; *m/z* 207) of (C) ZmTPS8 and (D) ZmTPS27. Samples were derived from vapor-phase extraction of *N. benthamiana* expressing P19 + HMGR either alone (-) or with the further addition of ZmTPS8 (*N.b.* TPS8) or ZmTPS27 (*N.b.* TPS27). Detectable ZmTPS8 hydrocarbon products are labeled in panel A. The retention time window (9.6- 12 minutes) was selected to include all relevant sesquiterpene hydrocarbons and alcohols. An inlay is provided for visual clarity of minor products between 10.5-10.8 min. Hydrocarbon analytes include (1) γ-cadinene (2) germacrene D (3) α-muurolene (4) unknown and (5) cadina-1(6),4-diene<trans->. Paired chromatographs in A and B as well as those in C and D are identically scaled. Heterologous expression of ZmTPS27 resulted in no clear sesquiterpene products (Panel B and D) while ZmTPS8 resulted in predominant sesquiterpene alcohols cubebol and copan-3-ol (panel A and C).

Figure S3

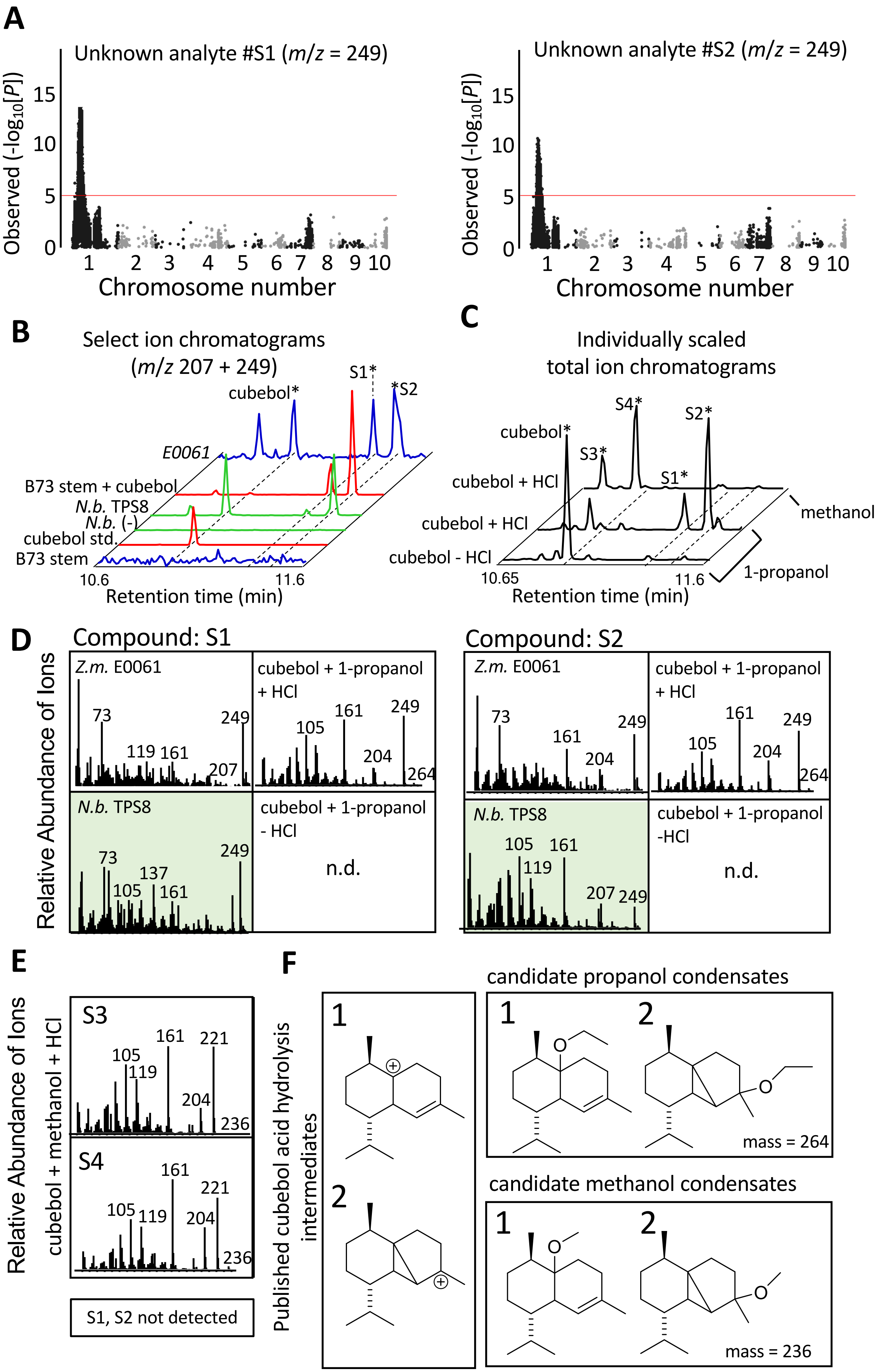

A
Unknown analyte #S1 (m/z = 249)
Unknown analyte #S2 (m/z = 249)
Observed (-log10[P])
Chromosome number
B
Select ion chromatograms
(m/z 207 + 249)
cubebol*
S1*
*S2
E0061
B73 stem + cubebol
N.b. TPS8
N.b. (-)
cubebol std.
B73 stem
10.6
11.6
Retention time (min)
C
Individually scaled
total ion chromatograms
cubebol*
S3*
S4*
S1*
S2*
cubebol + HCl
cubebol + HCl
cubebol - HCl
methanol
1-propanol
10.65
11.6
Retention time (min)
D
Compound: S1
Compound: S2
Relative Abundance of Ions
Z.m. E0061
cubebol + 1-propanol + HCl
N.b. TPS8
cubebol + 1-propanol - HCl
n.d.
E
Relative Abundance of Ions
cubebol + methanol + HCl
S3
S4
S1, S2 not detected
F
Published cubebol acid hydrolysis intermediates
candidate propanol condensates
mass = 264
candidate methanol condensates
mass = 236

Figure S3: Artifact formation of oxygenated sesquiterpenoid adducts through the acid solvolysis of ZmTPS8 products. (A) Manhattan plot of the M162W x B73 RILs (158 lines) using peak areas (*m/z* = 249) as a mapping trait for 2 unknown metabolites chromatographing after known sesquiterpene alcohols and highly correlated with ZmTPS8 products. Negative $\log_{10}$-transformed *P* values from the compressed mixed linear model are presented on the y-axis. The dashed line denotes the 5% Bonferroni-corrected threshold for 139,001 SNP markers, with the most statistically significant SNP located at positions (unknown analyte m/z 249 #S1, 55505430; unknown analyte m/z 249 #S2, 50708423) (B73 RefGen_V2) on chromosome 1. (B) GC-MS EIC chromatographs (*m/z* 207 + 249) from vapor phase extractions of maize tissues including B73 x M162W RIL E0061*;* wound treated B73 stem (max peak height = 2.5×10^4), B73 stem tissues mixed with 10 µg purified cubebol (B73 stem + cubebol (max peak height = 5.0×10^6), *N. benthamiana* heterologous expression of either P19 + HMGR alone [*N.b.* (-)], or the further additive addition of ZmTPS8 (*N.b.* TPS8; max peak height = 2.5×10^5). An unprocessed cubebol standard (cubebol std) was included for retention time (RT) comparisons. Dotted lines represent RT for pure cubebol (10.942 min,), analyte S1 (11.332 min, based on *E0061*), and analyte S2 (11.465 min, based on *E0061*). (C) GC-MS TIC chromatographs following headspace volatile extraction, performed at 100ºC, of cubebol incubated with either 1-propanol or methanol in an acidic (cubebol + HCl) or unaltered (cubebol – HCl) reaction environment. Dashed lines indicate the retention times of cubebol (10.942 min, based on cubebol - HCl in 1-propanol), S1 (11.332 min, based on cubebol + HCl in 1-propanol), and S2 (11.465 min, based on cubebol + HCl in 1-propanol). Two new major peaks, marked S3 and S4, were identified from headspace extraction of cubebol incubated in methanol + HCl. For visual clarity, chromatographs were scaled to emphasize the most abundant peaks. (D) EI mass spectra for analyte S1 (left, 11.332 min) and analyte S2 (right, 11.465 min) from B73 x M162W RIL E0061 in panel B (top left), cubebol incubated with acid-propanol in panel C (top right), *N. benthamiana* transiently expressing P19 + HMGR + TPS8 from panel B (bottom left, green), and cubebol incubated in 1-propanol without additional HCl from panel C (bottom right, n.d. indicates compound not detected). Major ions, including ions used for mapping in A, are labeled for visual clarity. Imperfect matching of all EI fragments is likely the result of complex samples and co-eluting compounds. The *m/z* 249 ion is a low abundance, rarely encountered and highly diagnostic marker in this study. (E) EI mass spectra for compounds S3 and S4 (panel C, cubebol + HCl in methanol). Major ions are labeled for visual clarity. (F) Proposed structures of compounds S1-4 based on published (left) cubebol solvolysis products following incubation in acidic solvent (Cornwell et al., 2000). Marked carbocation centers 1,2 are resolved through condensation with water in natural systems (Cornwell et al., 2000, Whitehead et al., 2022)(Kollner 2015 NEED..I don't know for sure the one intended here). As an explanation underlying the presence of additional analytes (S1 and S2; Panel A) in the association study, putative candidate structures for acid catalyzed solvolysis products are provided which could result from the substitution of propanol (S1, S2; top) and methanol (S3, S4; bottom) for H_2O in the established production schema of oxygenated cubebene derivatives.

Figure S4

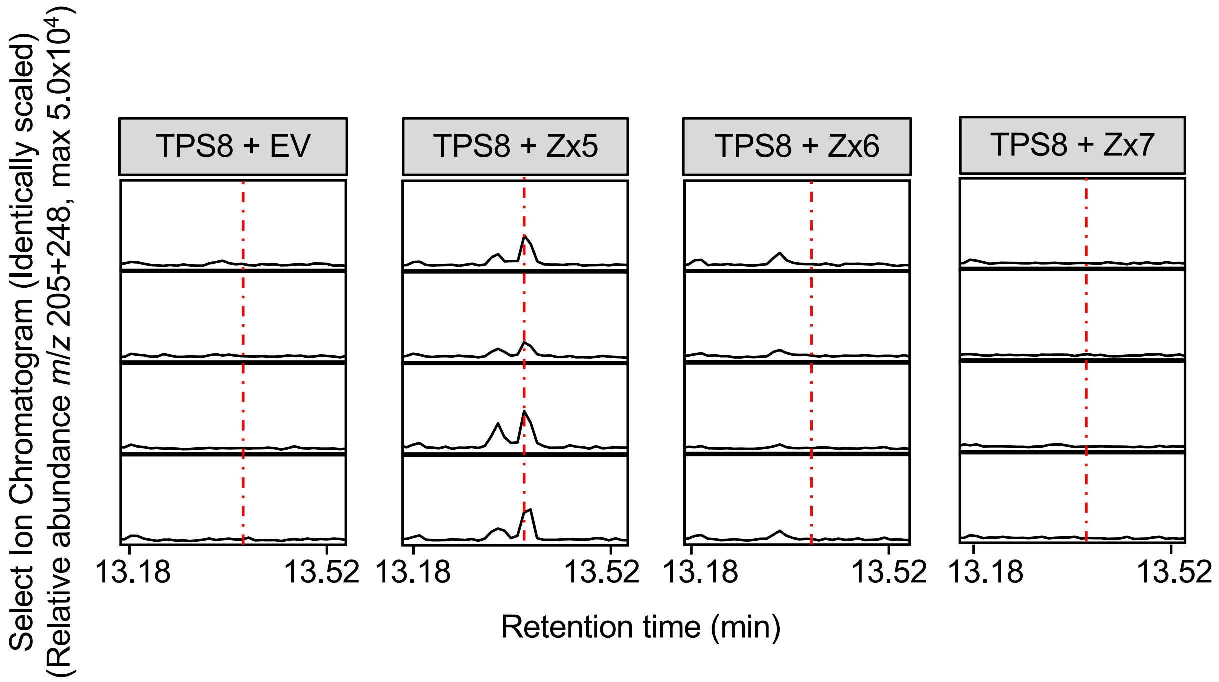

Figure S4: Production of analyte 9, an unknown sesquiterpene acid across individual *N. benthamiana* plants expressing ZmTPS8 specifically with ZmCYP71Z19. GC-MS EIC chromatograms (*m*/*z* 205 + 248) following vapor-phase extraction of *N. benthamiana* expressing ZmTPS8 in combination with pathogen-inducible CYPs Zx5 (ZmCYP71Z19), Zx6 (ZmCYP71Z18) and Zx7 (ZmCYP71Z16), with *ZmTPS8* + empty vector (EV) included as negative control. Each chromatograph represents an independent biological replicate. All chromatograms are identically scaled, with a cutoff abundance of 5.0 x 10^4. The dotted line represents retention time t = 13.373, consistent with the unknown sesquiterpene acid.

Figure S5

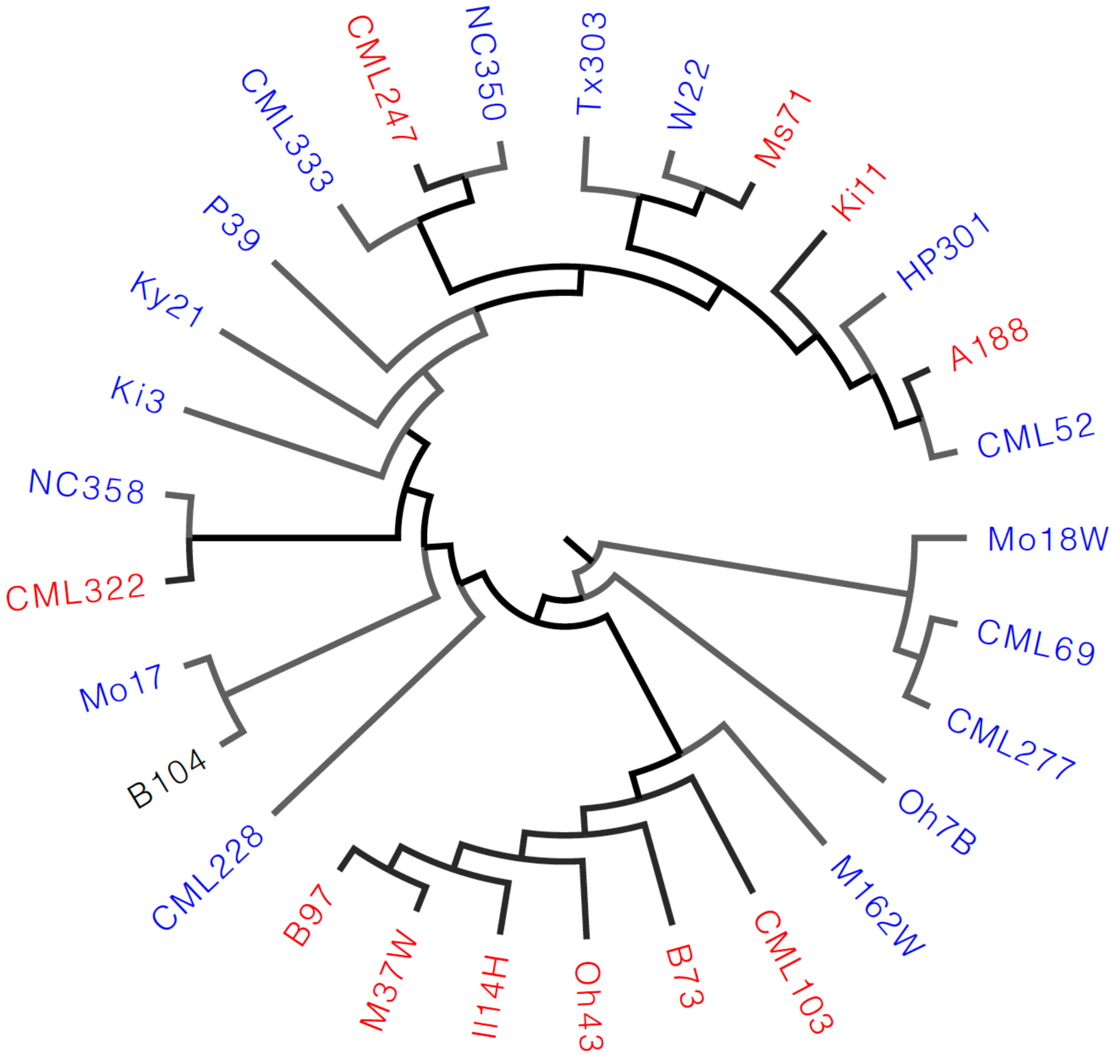

Figure S5: ZmTPS8 amino acid sequences from B73 and the four inbred lines with the highest average ZmTPS8 product accumulation form a distinct subclade. Protein sequences of ZmTPS8 gene models from diverse maize inbreds were obtained from MaizeGDB (Table S7). Protein sequences were aligned using famsa (v1.6.2) (Deorowicz et al., 2016) and clipped using ClipKIT (v1.1.5)(Steenwyk et al., 2020). IQ-TREE was used to select the best phylogenetic model and to generate the final tree (v2.1.4, default parameters and 1,000 bootstrap replications)(Kalyaanamoorthy et al., 2017, Nguyen et al., 2015) which was visualized and annotated with FigTree (http://tree.bio.ed.ac.uk/software/figtree/). **Red** inbred line names represent those where ZmTPS8 products were measurable and **Blue** inbred line names represent those with undetectable ZmTPS8 products (Fig. 1D). The B104 inbred line (black label) was included in this phylogeny yet not analyzed for ZmTPS8 products. B73 and the top 4 highest average ZmTPS8 product accumulating lines, namely M37W, IL14H, B97 and Oh43 (Fig. 1D) form a distinct subclade.
